# Body Fatness and Cardiovascular Health in Newborn Infants

**DOI:** 10.3390/jcm7090270

**Published:** 2018-09-11

**Authors:** Hasthi U. Dissanayake, Rowena L. McMullan, Yang Kong, Ian D. Caterson, David S. Celermajer, Melinda Phang, Camille Raynes-Greenow, Jaimie W. Polson, Adrienne Gordon, Michael R. Skilton

**Affiliations:** 1 Boden Institute of Obesity, Charles Perkins Centre, University of Sydney, Sydney, NSW 2006, Australia; hasthi.dissanayake@sydney.edu.au (H.U.D.); rowenamcm@gmail.com (R.L.M.); yangkong0310@hotmail.com (Y.K.); ian.caterson@sydney.edu.au (I.D.C.); melinda.phang@sydney.edu.au (M.P.); 2 Faculty of Medicine and Health, Charles Perkins Centre, The University of Sydney, Sydney, NSW 2006, Australia; david.celermajer@health.nsw.gov.au (D.S.C.); jaimie.polson@sydney.edu.au (J.W.P.); adrienne.gordon@sydney.edu.au (A.G.); 3 Royal Prince Alfred Hospital, Missenden Road, Camperdown, NSW 2050, Australia; 4 Sydney School of Public Health, The University of Sydney, Sydney, NSW 2006, Australia; camille.raynes-greenow@sydney.edu.au; 5 School of Medical Sciences & Bosch Institute, The University of Sydney, Sydney, NSW 2006, Australia

**Keywords:** newborn body fatness, cardiovascular disease, aortic intima-media thickness, autonomic function, cardiac structure, cardiac function

## Abstract

Birth weight is associated with cardiovascular disease, with those at both ends of the spectrum at increased risk. However, birth weight is a crude surrogate of fetal growth. Measures of body composition may more accurately identify high risk infants. We aimed to determine whether aortic wall thickening, cardiac autonomic control, and cardiac structure/function differ in newborns with high or low body fatness compared to those with average body fatness. 189 healthy singleton term born neonates were recruited and stratified by body fat percentiles (sex and gestation-specific). Infants with low body fat had higher aortic intima-media thickness (43 µm (95% confidence interval (CI) 7, 78), *p =* 0.02), lower heart rate variability (log total power, −0.5 (95% CI −0.8, −0.1), *p =* 0.008), and thicker ventricular walls (posterior wall thickness, 3.1 mm (95% CI 1.6, 4.6), *p* < 0.001) compared to infants with average body fatness. Infants with high body fat showed no differences in aortic intima-media thickness (−2 µm (95% CI −37, 33), *p =* 0.91) or cardiac structure compared to average body fatness, although stroke volume (−0.3 mL/kg (95% CI −0.6, −0.0), *p =* 0.003) and heart rate variability were lower (log total power, −0.8 (95% CI −1.1, −0.5), *p* < 0.001). The non-linear association of body fatness with heart rate variability was independent of birth weight. Infants born with low or high body fat have altered markers of cardiovascular health. Assessment of body fatness alongside birth weight may assist in identifying high risk individuals.

## 1. Introduction

Cardiovascular disease is the leading cause of death globally [1]. There is an extensive and consistent body of epidemiologic evidence supporting an association of birth weight with risk of adult cardiovascular disease, such that those at the lowest and highest birth weights are at highest risk [2,3,4,5]. 

Although cardiovascular disease presents clinically in adult life, the underlying disease processes commence in early life, can be modified by putative early life risk factors, and can be assessed using non-invasive techniques [6,7]. For example, small for gestational age newborns have increased aortic intima-media thickness (IMT), an age-appropriate marker of severity of the early stages of atherosclerosis [8]. Furthermore, changes in cardiac morphology and subtle impairments of cardiac function are present in newborns with fetal growth restriction, which persist through until at least late adolescence [9,10]. Underlying mechanisms may involve modification of the sympathetic and parasympathetic branches of the autonomic nervous system, which act together to regulate blood pressure and cardiovascular function starting in fetal life. Indeed, a number of studies show altered cardiac autonomic control, across the life course in people born small for gestational age [11,12]. 

Most evidence thus far has used birth weight as a surrogate marker of fetal growth and nutrition. However, birth weight is a crude measure of fetal growth, and is unable to differentiate constitutionally small infants who have met their genetic growth potential, from infants of the same weight but with pathological fetal growth restriction. Similarly, those who are large for gestational age may be either constitutionally or disproportionately large. It has been proposed that it is particularly those with restricted or excessive intrauterine growth who are at increased risk of adult cardiovascular disease [13]. One possible means by which to quantify newborn body composition is through the amount of lean and fat mass. Newborn lean mass primarily reflects genetic and familial determinants such as parental size [14], whereas fat mass is more variable, and likely relates to the intrauterine environment, particularly during the final trimester [14,15,16].

Accordingly, we sought to determine whether aortic IMT, cardiac autonomic control and cardiac structure and function differ in newborns with high or low body fatness, when compared to those with average body fatness. Furthermore, we sought to determine whether body fatness is a better predictor than birth weight of any such alterations. 

## 2. Methods

### 2.1. Participants

Infants were recruited from the postnatal wards and the neonatal unit at the Royal Prince Alfred Hospital, Sydney. Eligible subjects were well singleton newborns between 37 and 42 completed weeks gestation that had undergone routine body composition measurements. Exclusions were major congenital abnormalities and ongoing need for respiratory support. Recruitment was stratified according to published gestation and sex specific body fat percentiles (≤10th, >10th to ≤25th, >25th to ≤50th, >50th to ≤75th, >75th to ≤90th, >90th) [17], with an approximate sex-balance in each group. Gestational age was calculated from first trimester ultrasounds. Recruitment details and participant flow is shown in Supplemental Figure S1.

This study was conducted in accordance with the Declaration of Helsinki and the protocol was approved by the Sydney Local Health District ethics committee (protocol X14-0356 & HREC/14/RPAH/478). Participation was voluntary, and informed consent was obtained from all parents.

### 2.2. Body Composition and Anthropometry

Body composition was measured in infants in the first 24 h postpartum using air-displacement plethysmography (PEA POD^®^, COSMED Inc., Chicago, IL, USA), as part of their routine clinical management. Air-displacement plethysmography accurately measures body volume, and thus body density, which can be used to calculate fat mass and fat-free mass [18,19,20]. Birth weight, length and head circumference were measured as part of routine practice. Weight was measured with the integrated PEA POD^®^ scales to the nearest gram, head circumference using disposable infant paper tapes to 0.1 cm, and length to the nearest 0.1 cm (Easy-Glide Bearing Infantometer, Perspective Enterprises, Portage, MI, USA).

### 2.3. Maternal and Infant Demographics

Demographic data, health status, pregnancy and delivery details on mothers and newborns were collected directly from mothers using standardised questionnaires and medical records.

### 2.4. Aortic Intima-Media Thickness

The abdominal aorta was imaged by ultrasound (EPIQ 5, Philips Medical Systems, Bothell, WA, USA), with a high-frequency linear probe (18–5 MHz). The image was optimised to focus on the dorsal wall of a straight, non-branching segment. Aortic IMT was measured offline using validated semi-automated edge-detection software (Carotid Analyzer, Medical Imaging Applications, Coralville, IA, USA) by a blinded observer (Y.K.). Maximum aortic IMT was calculated as the average of maximum thickness from a minimum of 3 analyzed frames, and was our primary outcome given the relevance of this measure to the pathophysiology of early atherosclerosis, particularly the focal nature of early lesions [8,21,22]. Analyses of 20 randomly selected scans were repeated by same observer at least one month apart, with intra-class correlations of 0.91 (mean IMT) and 0.90 (maximum IMT).

### 2.5. Heart Rate Variability

A 3-lead electrocardiogram (ECG) was recorded continuously for 15 min whilst the infants were sleeping in a supine position. The ECG analogue output was digitised at 500 Hz and acquired (Powerlab, ADInstruments, Sydney, Australia). Infant behaviour was observed closely. Any periods of wakeful activity were noted, and these periods removed from subsequent analysis. Analysis of heart rate and heart rate variability (HRV) were performed using LabChart (HRV 1 module, version 7, ADInstruments, Sydney, Australia) on up to three (average 2.75) R-R interval epochs of exactly 4 min. Peak detection on ECG was used to create RR sequences. Time domain measures of HRV included the standard deviation of the normal-to-normal (NN) RR intervals as a measure of overall variability (SDNN), and two short-term measures: standard deviation of change in successive NN intervals (SD∆NN), and the root mean square of successive differences in NN interval (RMSSD) [23]. Spectral bands for frequency domain analysis of HRV were investigated in the range of 0 to1.1 Hz: low frequency at 0.04 to 0.15 Hz, and high frequency at 0.15 to 1.1 Hz [24]. The high frequency band was based on respiratory rates of 0.5 to 1 Hz [24]. VLF was not determined due to the short sampling times.

### 2.6. Cardiac Ultrasound

Two-dimensional cardiac ultrasound was performed with a Philips EPIQ 5 and S12-4 Phased Sector Array transducer using a standardised protocol, from day 2 after birth to allow for postpartum cardiovascular transition. Images were stored and blinded offline analysis performed with Xcelera software V (Philips Medical Systems, Veenpluis, The Netherlands). Detailed cardiac ultrasound methods are described in Supplementary Materials. Blinded analyses were performed on two separate occasions at least one month apart for 20 randomly selected participants to determine intra-observer variability. Intra-class correlations ranged from 0.75 to 0.98 (Supplemental Table S1).

### 2.7. Statistical Analysis

Continuous data are expressed as mean (SD) and categorical data as *n* (%). Data were tested visually for normality, and log transformations applied as required. Log transformed data are presented as the median (interquartile range). Maximum aortic IMT was our prespecified primary outcome, with the primary analysis being multivariable linear regression to compare high body fat ((HBF) > 90th percentile) and low body fat ((LBF) ≤ 10th percentile) infants with average body fatness (>25th to ≤75th percentile). An *a priori* power calculation indicated that 30 LBF infants and 40 average BF infants, would provide 83% power to detect a difference in aortic IMT of 50 µm, assuming standard deviation of 70, at 2*p* < 0.05. Post hoc analysis was undertaken to directly compare the high body fat and low body fat infants. Chi–Square tests were utilised for categorical data. Nonlinear associations were determined by use of quadratic terms in multivariable regression models that included participants across the entire body fat spectrum, adjusted for birth weight. Statistical significance was inferred at 2*p* < 0.05. Statistical analysis was performed using IBM SPSS Statistics for Windows, version 23 (IBM Corp., Armonk, NY, USA).

## 3. Results

### 3.1. Participant Characteristics

Mothers of infants with LBF were more likely to identify as South Asian, and less likely as Caucasian, compared to mothers of infants with average body fatness. Maternal body mass index was higher in mothers who gave birth to infants with HBF, who were also more like to deliver by caesarean section (Table 1). The prevalence of maternal smoking and gestational diabetes did not differ between test groups.

LBF infants were lighter, shorter and had reduced head circumference compared to infants with average body fatness, whereas HBF infants were heavier, longer and had a larger head circumference (Table 1).

Maternal and infant characteristics for those infants with body fat percentage between the 10th to 25th percentile and 75th to 90th percentile, are provided in Supplemental Table S2.

Unadjusted values for aortic IMT, autonomic activity and cardiac structure and function by body fatness group are shown in Supplemental Tables S3 and S4.

### 3.2. Aortic Intima-Media Thickness

**Figure 1:**
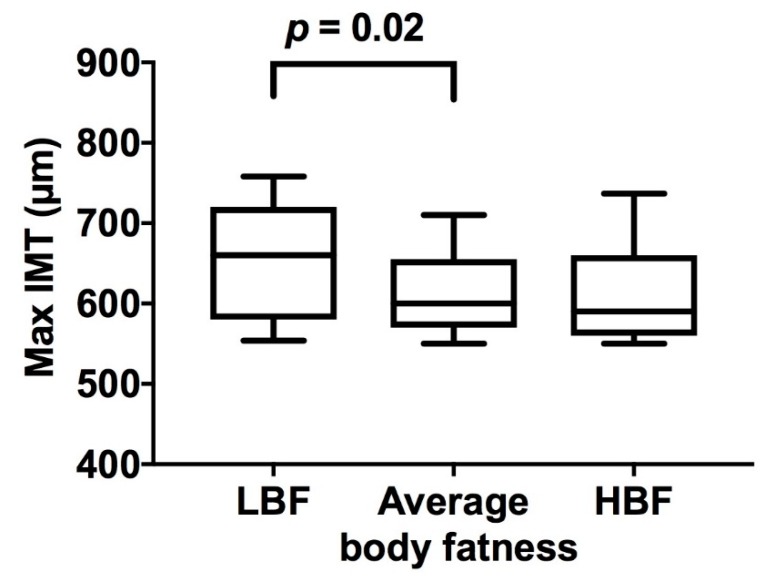
Effect of infant body fatness on maximum aortic intima-media thickness. LBF: low body fat (≤10th BF%); HBF: high body fat (>90th BF%); average body fatness (>25th to ≤75th BF%). Box, line, and error bars represent 10th, 25th, 50th (median), 75th and 90th percentiles.

Maximum aortic IMT was greater in LBF infants, but not HBF infants, when compared to average body fatness infants (Figure 1 and Table 2). Across the entire body fat percent spectrum, there was some evidence of a non-linear association between infant body fatness and aortic IMT (*p =* 0.07, adjusted for gestational age and sex), although body fatness only accounted for 3.7% of the variance in aortic IMT. In contrast, there was a strong non-linear association of birth weight with aortic IMT (*p* < 0.001), with birth weight explaining 13.3% of the variance in aortic IMT. Indeed, aortic IMT was markedly higher in those born at either end of the birth weight spectrum (birth weight ≤10th percentile: 83 µm (95% CI 49, 118), *p* < 0.001; birth weight >90th percentile: 43 µm (95% CI 4, 82), *p =* 0.03; both compared to birth weight between the 25th and 75th percentile, adjusting for gestational age and sex). The association of birth weight ≤10th percentile with aortic IMT was independent of percent body fatness (*p* < 0.001), but the association of birth weight >90th percentile was not independent of body fatness (*p =* 0.20).

### 3.3. Heart Rate Variability

Heart rate was similar across body fat percentiles (Table 2). Measures of overall HRV, in the time (SDNN) and frequency domain (total power) were lower in LBF and HBF than infants with average body fatness. These associations remained significant after adjustment for gestational age and sex (e.g., for log total power: LBF, −0.5 (95% CI −0.8, −0.1), *p =* 0.008; HBF, −0.8 (95% CI −1.1, −0.5), *p* < 0.001; both compared to average body fatness infants) (Table 2).

Furthermore, SD∆NN and RMSSD, both short-term time domain measures, and the HF and LF power measures were all lower in HBF infants compared to average body fatness infants (Table 2).

Across the entire body fat percent spectrum, there was a non-linear association between infant body fat percent and overall HRV (total power, *p =* 0.001, adjusted for gestational age and sex), which was independent of birth weight (*p =* 0.01). Body fat percent accounted for 8.6% of the variance in overall HRV. In comparison, birth weight accounted for 8.2% of the variance in overall HRV. In a model which included birth weight, the introduction of body fat percent accounted for an additional 4.7% of the variance in overall HRV.

### 3.4. Cardiac Structure and Function

LBF infants had significantly increased septal and posterior wall thicknesses and left ventricular chamber dilatation compared to infants with average body fatness (Table 2). Cardio-physiological parameters were similar between LBF and average body fatness infants (Supplemental Table S5). Infants with HBF had increased ventricular base to apex length, and lower left ventricular stroke volume compared to infants with average body fatness (Table 2 and Supplemental Table S5), with no other differences observed in cardiac structure or measures of systolic or diastolic function.

There were no significant associations, linear or non-linear, between cardiac measures and body fat percent across the entire spectrum of body fat measures (results not shown). Six infants had a small ventricular septal defect or patent ductus arteriosus, which were asymptomatic with minimal shunt. Sensitivity analyses excluding these infants did not change the results.

## 4. Discussion

Newborn body fatness maybe a more sensitive marker of fetal growth than birth weight, in identify those at high risk of cardiovascular disease. Our findings indicate that infants with LBF have increased aortic IMT, reduced cardiac autonomic control, thicker ventricular walls, and larger cardiac chamber size. Infants with HBF also showed reduced cardiac autonomic control, although this was accompanied by a different cardiac phenotype, specifically an isolated reduction in stroke volume that was compensated by an increase in heart rate, without cardiac or vascular structural adaptation. Furthermore, the association of body fatness with cardiac autonomic control was independent of birth weight, and stronger than the equivalent association of birth weight with cardiac autonomic control. However, the opposite was true for aortic IMT, with birth weight being more strongly associated with aortic IMT, and independent of percent body fat. This suggests that low and high body fatness and weight at birth are each accompanied by specific cardiovascular phenotypes that may mechanistically contribute to distinctly different cardiovascular risk pathways.

Precise identification of individuals at high risk of cardiovascular disease on the basis of early life risk factors remain a priority. We and others have previously shown that individuals with impaired fetal growth, based on birth weight percentiles, have increased aortic IMT during infancy [7,8,25,26] and increased carotid IMT as adults [27].

We hypothesised that high and low body fatness at birth would better identify infants with early evidence of poor cardiovascular health, and in particular, increased aortic IMT. However, we found that at both ends of the growth spectrum, birth weight is a stronger predictor of aortic IMT. This may relate to the timing of fat deposition in the fetus, principally in the third trimester. As such it may be that aortic IMT in the infant, as a structural marker of vascular health that develops over time, may be more strongly influenced by net fetal growth throughout pregnancy. It may be that an association of infant body fatness with aortic IMT will develop over time, consistent with a previous report of an association of skinfold thickness at birth, as a marker of adiposity, with aortic IMT at 6 weeks of age [28]. The implications for risk of heart disease in adulthood associated with a 50 μm higher aortic IMT in infancy are not known. However, the magnitude of the observed effect is consistent with previous studies by ourselves by others [8,22], and aging-related increases in aortic IMT through until early adulthood are about 2.5× the magnitude of those for carotid IMT [29]. This tentatively suggests that a 50 µm higher aortic IMT may infer a 25–37% higher risk of myocardial infarction [30].

In contrast, body fatness was more strongly associated with autonomic activity than birth weight. Autonomic activity is a functional mechanistic component of cardiovascular control and may respond more rapidly and dynamically to the short-term fetal growth disturbances in the third trimester that are captured by differences in newborn body fatness. Previous studies have described altered autonomic functions in people born with low birth weight, from infancy through to adult life [12,31,32]. Reduced HRV may indicate a reduced ability to adapt to internal and external stimuli making these individuals mechanistically susceptible to future hypertensive and cardiac diseases, particularly via autonomic pathways.

Interestingly we found that infants with HBF also had lower overall HRV and overall reduction in parasympathetic modulation to the heart. This finding is consistent with previous studies showing reduced HRV in older children and adults with increased adiposity [33,34,35]. We did not seek to determine the specific mechanistic pathways that link infant adiposity to reductions in HRV, although they may involve fetal glucose, insulin and adipocytokines. However, increased adiposity is a related yet distinct pathway consistent with predisposition to developing hypertension. It is unclear whether altered cardiac autonomic function persists into adulthood, however it may contribute to a proportion of the risk of developing later cardiovascular disease.

Furthermore, these reductions in HRV in infants with LBF or HBF may contribute to the higher risk of stillbirth at the extremes of the fetal growth spectrum, although further work would be required to develop and test this hypothesis.

The most commonly reported structural cardiac adaptation that accompanies fetal growth restriction is a globular ventricle as assessed by sphericity index [6,7]. This adaptive structural change has been demonstrated to be related to the severity of growth restriction as defined by body weight and fetal Doppler assessment [9]. Whilst we did not observe a significant difference in sphericity index in the LBF group, we found that infants with LBF have thicker ventricular walls and larger chamber size, consistent with the structural adaptations previously described, albeit within the normal range. Proposed mechanisms linking growth restriction and altered cardiac geometry include compensatory remodelling secondary to increased myocardial workload in utero, compounded by the effects of chronic hypoxia and undernutrition on the developing myocardium. Indeed, growth restricted infants have been shown to have increased aortic wall stiffness and raised peripheral vascular resistance [7,36], which along with increased placental vascular resistance [37], contribute to raised afterload against which the immature myocardium must pump. Studies describe subtle reductions in diastolic function [6,36,38] and these cardiac functional alterations are proposed to be secondary to increased afterload and altered geometry affecting ventricular relaxation, and are also related to severity of growth restriction [9]. This hypertrophic phenotype of fetal origin persists into mid-childhood and adulthood [9,10,39], albeit more subtle with age. Whether the associations of body fat percent with this cardiac phenotype will persist, strengthen, or weaken over time remains unknown.

A key strength of our study is that we investigated infants at a very early age, on average 2–3 days, allowing us to by-and-large exclude postnatal exposures. We also have consent to follow up these infants to determine the effect of newborn body composition on risk of later disease. Furthermore, we did not focus on a single measure of cardiovascular health in isolation, but rather have taken a broader approach to cardiovascular health assessment through the use of complementary indices that cover a range of the pathophysiology involved in early cardiovascular disease.

We acknowledge the limitations in this study. Not all cardiovascular measures were undertaken in all participants owing largely to participant compliance and time restrictions prior to leaving hospital, prioritizing our primary outcome, aortic IMT. This study has a similar sample size to that of other work in the literature, albeit relatively small. Future studies may wish to confirm these findings in larger cohorts, including in the offspring of healthy women without complications of pregnancy such as gestational diabetes or hypertension. The body fat percentile reference data were established in a largely Caucasian population [17], which differs from our mixed ethnic population. Adjusting for ethnicity did not materially affect the associations seen between body fatness, aortic IMT, cardiac autonomic function or cardiac structure and function. Nonetheless it remains controversial as to whether ethnic differences in cardiometabolic risk relate to differences in fetal growth [40]. Although autonomic measurements were taken during sleep, studies have shown that HRV differs according to sleep state (active vs. quiet sleep). Finally, it is possible that multiple testing across various outcomes without adjustment for multiple testing may have contributed to the likelihood of false positives. However, our findings for birth weight are consistent with those previously described by ourselves and others in infancy and childhood, as such suggesting that our novel findings concerning body fatness are unlikely to be a chance finding.

## 5. Conclusions

Atherosclerotic and hypertensive diseases have distinct and shared pathophysiology. Fetal programming of these diseases is established from extensive epidemiology, although accurate identification of high risk infants, and the underlying life course mechanisms involved, remain poorly described. Our key finding is that low and high body fatness, and low and high birth weight, are selectively and differentially associated with markers of cardiac and vascular health in newborns. Importantly, body fatness and birth weight appear to provide complementary information for identification of infants with poorer cardiovascular health markers. This suggests that assessment of body fatness may play a role, alongside birth weight, in identifying individuals at high risk of cardiovascular and hypertensive diseases. Future studies may seek to develop distinct life course prevention strategies that counter the identified pathophysiology, as a precision medicine strategy to reduce risk of hypertension and atherosclerotic-vascular disease in people exposed to an adverse intrauterine environment [41,42].

## Figures and Tables

**Table 1:** Maternal and infant characteristics.

	Average BF	Low BF	Low BF vs. Average BF *p* Value	High BF	High BF vs. Average BF *p* Value	Low BF vs. High BF *p* Value
Maternal Characteristics						
Age, years	33 (4)	32 (4)	0.28	35 (5)	0.14	0.03
Pre-pregnancy BMI, kg/m^2^	23 (5)	22 (3)	0.34	24 (4)	0.17	0.006
Pre-pregnancy weight, Kg	62 (12)	57 (9)	0.04	65 (11)	0.23	0.001
Height, cm	166 (6)	161 (7)	0.002	167 (10)	0.92	0.01
Weight at first antenatal visit, kg	63 (12)	58 (9)	0.04	67 (7)	0.92	<0.001
Gestational Diabetes, *n* (%)	11 (19)	5 (15)	0.78	4 (13)	0.38	0.84
Preeclampsia, *n* (%)	2 (3)	2 (6)	0.61	2 (7)	0.60	0.92
Hypertension in pregnancy, *n* (%)	2 (3)	1 (3)	0.93	1 (3)	0.74	0.95
Maternal smoking, *n* (%)	1 (2)	3 (10)	0.18	0 (0)	0.26	0.13
Ethnicity, *n* (%)						
Asian	12 (20)	5 (16)	0.05	4 (13)	0.83	
Caucasian	40 (68)	14 (45)		23 (77)		
Middle Eastern	1 (2)	2 (7)		0 (0)		0.06
South Asian	3 (5)	7 (23)		1 (3)		
Other	3 (3)	3 (10)		2 (7)		
Mode of birth, *n* (%)						
Vaginal	36 (61)	20 (61)	0.99	13 (43)	0.03	
Instrumental vaginal	13 (22)	7 (21)		4 (13)		0.09
Caesarean	10 (17)	6 (18)		13 (43)		
Labour						
Spontaneous	35 (59)	17 (52)	0.76	10 (33)	0.001	
Induced	19 (32)	13 (39)		7 (23)		0.008
No Labour	5 (9)	3 (9)		13 (43)		
Infant Characteristics						
NICU admissions, *n* (%)	3 (6)	4 (13)	0.42	2 (7)	0.60	0.41
Postnatal age, days	2 (1)	3 (2)	0.19	2 (2)	0.69	0.42
Gestational age, weeks	39.3 (1.2)	38.7 (0.9)	0.01	39.2 (1.3)	0.61	0.09
Sex, female/male	33/26	18/15	1.00	15/15	0.66	0.72
Birth weight, g	3386 (412)	2916 (360)	<0.001	3983 (435)	<0.001	<0.001
Apgar score at 1 min	8.7 (0.84)	8.2 (1.7)	0.14	8.9 (0.57)	0.26	0.05
Apgar score at 5 min	9.0 (0.23)	8.8 (0.58)	0.07	9.0 (0.18)	0.30	0.17
Length, cm	50 (2)	48 (2)	<0.001	51 (2)	<0.001	<0.001
Head circumference, cm	35 (1)	34 (1)	0.004	36 (1)	<0.001	<0.001
Body fat, %	11 (2)	4 (2)	<0.001	18 (2)	<0.001	<0.001

Data are presented as mean (SD) for continuous variables using independent student *t*-tests and No. (%) for categorical data, using chi-square tests between groups. LBF, low body fat; HBF, high body fat; BMI, body mass index; NICU, neonatal intensive care unit. Average body fatness, >25th to ≤75th BF%, (*n =* 59); Low body fatness, ≤10th BF% (*n =* 33); High body fatness, >90th BF% (*n =* 30); except for maternal BMI *n =* 57, *n =* 32, *n =* 26; maternal height, *n =* 57, *n =* 32, *n =* 26; pre-pregnancy weight *n =* 55 (average body fatness), *n =* 31 (Low BF); and weight at first antenatal visit *n =* 27 (High BF).

**Table 2:** Infant body fatness and aortic intima-media thickness, heart rate variability and cardiac structure.

	Low BF (Relative to Average BF)		High BF (Relative to Average BF)		High BF (Relative to Low BF)	
	*β* (95% CI)	*p* Value	*β* (95% CI)	*p* Value	*β* (95% CI)	*p* Value
Aortic IMT	*n =* 31		*n =* 30			
Maximum IMT, µm	43 (7, 78)	0.02	−2 (−37, 33)	0.91	−49 (−91, −8)	0.02
HRV (frequency domain)	*n =* 25		*n =* 26			
Ln Total Power	−0.5 (−0.8, −0.1)	0.01	−0.8 (−1.1, −0.5)	<0.001	−0.3 (−0.7, 0.1)	0.16
Ln LF	−0.3 (−0.7, 0.1)	0.18	−0.8 (−1.1, −0.4)	<0.001	−0.4 (−0.9, −0.0)	0.05
Ln HF	−0.2 (−0.7, 0.3)	0.51	−1.0 (−1.4, −0.4)	<0.001	−0.7 (−1.2, −0.1)	0.02
Ln LF: HF	−0.2 (−0.6, 0.2)	0.38	0.3 (−0.1, 0.8)	0.15	0.5 (−0.1, 1.0)	0.12
HRV (time domain)	*n =* 25		*n =* 26			
HR, bpm	−0.5 (−9.3, 8.4)	0.92	4.4 (−4.3, 13.1)	0.32	3.0 (−7.1, 13.2)	0.55
Mean NN, ms	11.4 (−20.9, 43.8)	0.48	−18.3 (−50.0, 13.4)	0.25	−23.1 (−60.9, 14.8)	0.23
SDNN, ms	−7.1 (−13.2, −1.0)	0.02	−11.2 (−17.2, −5.3)	<0.001	−3.0 (−9.7, 3.6)	0.36
Ln SD∆NN	−0.1 (−0.4, 0.2)	0.52	−0.5 (−0.7, −0.2)	0.02	−0.3 (−0.6, 0.0)	0.06
Ln RMSSD	−0.1 (−0.4, 0.2)	0.39	−0.4 (−0.7, −0.1)	0.004	−0.2 (−0.5, 0.1)	0.14
Cardiac structure	*n =* 22		*n =* 23			
LV Base to apex length, mm	−1.3 (−3.1, 0.3)	0.11	2.1 (0.3, 3.8)	0.02	3.8 (1.2, 6.2)	0.01
LV Diameter, mm	−0.9 (−2.0, 0.3)	0.13	0.1 (−1.1, 1.3)	0.89	1.0 (−0.8, 2.7)	0.27
LV Sphericity index	0.1 (−0.1, 0.2)	0.46	0.2 (−0.0, 0.3)	0.09	−0.0 (−0.2, 0.2)	0.93
Septal wall thickness mm/BSA	2.3 (0.5, 4.1)	0.01	−0.0 (−1.8, 1.7)	0.99	−2.5 (−4.9, 0.1)	0.06
Posterior wall thickness mm/BSA	3.1 (1.6, 4.6)	<0.001	−1.0 (−2.4, 0.5)	0.18	−4.1 (−5.8, −2.4)	<0.001
End-diastolic dimension mm/BSA	5.5 (1.4, 9.7)	0.01	−3.0 (−7.1, 1.0)	0.14	−9.0 (−14.0. −4.0)	0.001
Relative wall thickness	0.03 (−0.00, 0.7)	0.08	−0.01 (−0.04, 0.03)	0.71	−0.04 (−0.08, 0.01)	0.09
RV Base to apex length, mm	−0.8 (−2.6, 1.0)	0.36	2.0 (0.4, 3.7)	0.02	2.9 (0.5, 5.4)	0.02
RV Diameter, mm	−0.7 (−1.7, 0.4)	0.19	0.4 (−0.5, 1.4)	0.38	1.2 (0.1, 2.2)	0.03
RV Sphericity index	0.0 (−0.1, 0.2)	0.87	0.1 (−0.1, 0.2)	0.23	0.3 (−0.2, 0.7)	0.24

Values are unstandardized β-regression coefficients (95% CI) from multivariable models, adjusted for sex and gestational age. Reference groups are Average BF and Low BF, as indicated in column header. Ln, log transformed data. Average body fatness, >25th to ≤75th BF%; low body fatness, ≤10th BF%; high body fatness, >90th BF%. Average body fatness; *n =* 49 (aortic IMT), *n =* 39 (HRV), *n =* 46 (cardiac structure). IMT, intima-media thickness; HRV, heart rate variability; LF, low frequency; HF, high frequency; LF: HF, low frequency/high frequency ratio; HR; heart rate, mean NN; mean of N wave to N wave variation normal; SDNN, the mean of the standard deviation of all normal RR intervals; SD∆NN, SD change in NN; RMSSD, square root of the mean squared differences of successive NN intervals; LV, left ventricle; RV, right ventricle; BSA, body surface area.

## Supplementary Materials

The following are available online at http://www.mdpi.com/2077-0383/7/9/270/s1, Figure S1: Study flow, Table S1: Intra-class correlation, Table S2: Maternal and infant characteristics for infants in >10th and 25th and >75th and ≤90th groups, Table S3: Aortic intima-media thickness and heart rate variability across body fat percentiles, Table S4: Cardiac structure and function across body fat percentiles, Table S5: Indices of cardiac function in infants with low, high and average body fatness.
