# Supplementary material for: Body Fatness and Cardiovascular Health in Newborn Infants
Source: J Clin Med. 2018 Sep 11;7(9):270. doi: 10.3390/jcm7090270 (PMC6162858; doi:10.3390/jcm7090270)
Supplement: Supplementary file 1 [file jcm-07-00270-s001.pdf]

Supplemental Methods

Cardiac dimensions: Left and right ventricle base to apex length and diameter were measured in apical 4 chamber view at end-diastole, and sphericity index calculated as length/diameter. Left ventricular wall thicknesses were measured at end-diastole just distal to the mitral leaflets in M-mode from a parasternal long axis view. Relative wall thickness was posterior wall thickness + septal thickness/left ventricular end diastolic dimension. Wall thicknesses were indexed to body surface area as calculated by the Haycock formula [1].

***Systolic function*:** Fractional shortening was measured in M-mode from a parasternal long axis view, and calculated as end-diastolic diameter – (end-systolic diameter/end-diastolic diameter). Ascending aortic velocity time integral was measured from an apical 5 chamber view with pulsed-wave Doppler. Internal aortic diameter was measured at parasternal long axis view where the aortic valve leaflets were clearly visualized. Stroke volume was calculated as aortic area x heart rate, and left ventricular output as aortic area x velocity time integral x heart rate. Mitral and tricuspid annular plane systolic excursion were measured using M-mode through respective lateral valve leaflets from apical 4 chamber view. Peak systolic myocardial velocity was measured with tissue Doppler imaging at the lateral and septal mitral and lateral tricuspid valves utilising pulsed-wave Doppler from the apical 4 chamber view.

***Diastolic function*:** Ventricular inflow was measured using pulsed-wave Doppler of the atrioventricular valves from an apical 4 chamber view. Peak velocities of the E and A waves were measured and used to calculate the E:A ratio. Isovolumetric relaxation time was measured from an apical view, with the sample volume placed to simultaneously record both mitral and aortic flow, and calculated as the end of flow through the aortic valve to the beginning of flow through the mitral valve. Peak diastolic myocardial velocities (E' and A') were measured with tissue Doppler imaging at the lateral and septal mitral and lateral tricuspid valves utilising pulsed-wave Doppler from the apical 4 chamber view. Lateral and septal E: E' were calculated.

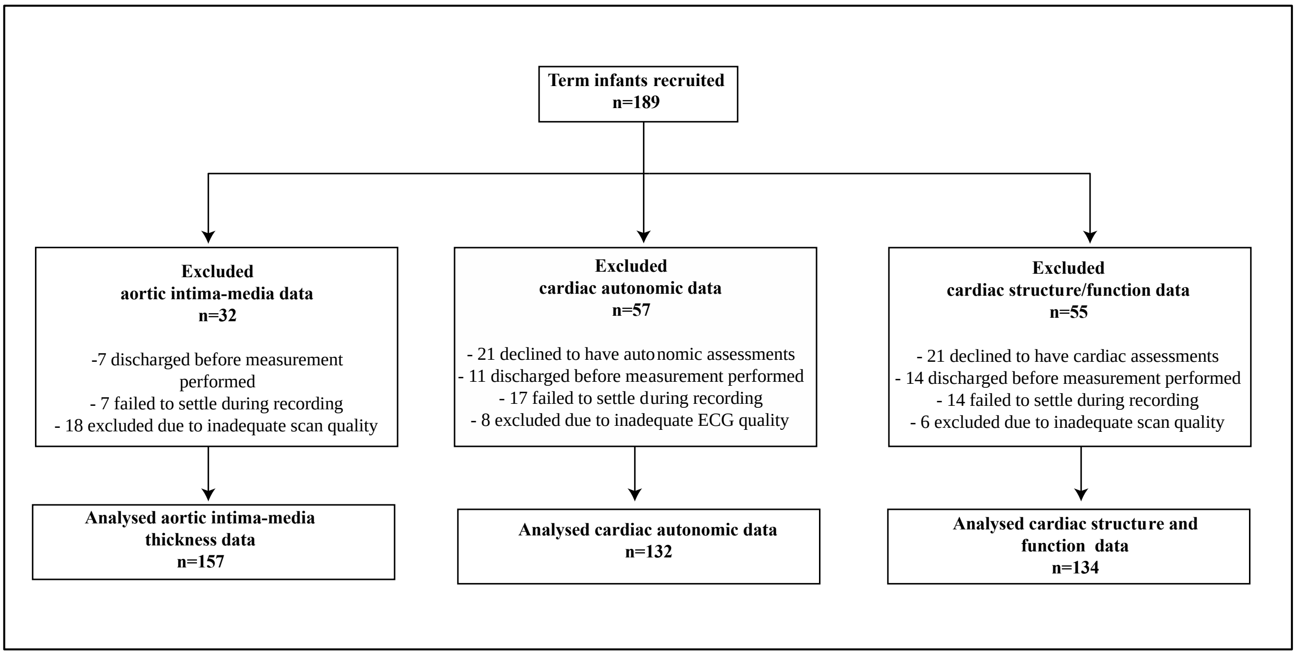

Figure S1. Study Flow.

Table S1. Intra-class correlation.

	Coefficient of variation	ICC (95% CI)
Left ventricle end-diastolic diameter	3.9	0.9 (0.8,1.0)
Left ventricle end-systolic diameter	4.0	0.8 (0.5,0.9)
Ascending aorta velocity time integral	3.7	1.0 (1.0,1.0)
Left ventricular output	15.1	0.8 (0.6,1.0)

Table S2. Maternal and infant characteristics for infants in >10th and ≤25th and >75th and ≤90th groups.

	(>10th and ≤25th BF%) (n=25)	(>75th and ≤90th BF%) (n=29)
Maternal Characteristics		
Age, years	33 (5)	33 (4)
Maternal pre-pregnancy BMI, Kg/m^2	24 (4)	24 (4)
Pre-pregnancy weight, Kg	63 (14)	68 (13)
Height, cm	164 (5)	166 (7)
Weight at first antenatal visit, kg	62 (13)	66 (11)
Gestational Diabetes Mellitus, n (%)	2 (8)	5 (17)
Preeclampsia, n (%)	1 (4)	0 (0)
Hypertension in pregnancy, n (%)	1(4)	0 (0)
Maternal smoking, n (%)	0 (0)	0 (0)
Ethnicity, n (%)		
Asian	6 (25)	5 (17)
Caucasian	13 (54)	21 (72)
Middle Eastern	1 (4)	1 (3)
South Asian	3 (13)	2 (7)
Other	0 (0)	0 (0)
Mode of birth, n (%)		
Vaginal	14 (56)	17 (59)
Instrumental vaginal	6 (24)	3 (10)
Caesarean	5 (20)	9 (31)
Labour		
Spontaneous	15 (60)	16 (55)
Induced	8 (32)	7 (24)
No Labour	2 (8)	6 (21)
Infant Characteristics		
NICU admissions n (%)	1 (4)	2 (7)
Postnatal age, days	2 (1)	2 (1)
Gestational age, weeks	39 (1)	39 (1)

Sex, female/male)	12 / 13	16 /13
Birth weight, g	3057 (300)	3653 (341)
Length, cm	50 (2)	50 (2)
Head circumference, cm	34 (1)	35 (1)
Body fat (%)	7 (1)	15 (2)

Data are presented as mean (SD) for continuous variables and No. (%) for dichotomous variables. BMI, body mass index; NICU, neonatal intensive care unit. >10th and ≤25th BF%, n = 25; >75th and ≤90th BF%, n = 29, except maternal pre-pregnancy BMI n = 21, n = 26; maternal height, n = 21, n = 27; pre-pregnancy weight n = 22, n= 28 respectively and >75th and ≤90th BF%, n = 27 weight at first antenatal.

Table S3. Aortic intima-media thickness and heart rate variability across body fat percentiles.

	**Average body fatness (>25th to ≤75th BF%)**	**LBF (≤10th BF%)**	**LBF vs Average BF *P* value**	**HBF (>90th BF%)**	***HBF vs Average BF P* value**	***LBF vs HBF P* value**
**Aortic IMT**	**n=49**	**n=31**		**n=30**		
Maximum IMT, μm	614 (71)	654 (89)	.03	610 (74)	.83	.04
Mean IMT, μm	551 (62)	558 (64)	.62	536 (66)	.29	.18
**Heart rate variability**	**n=39**	**n=25**		**n=26**		
Frequency domain						
*Total Power, ms^2	1541 (1313)	821 (1226)	.01	749 (553)	<.001	.09
*LF, ms^2	369 (384)	258 (318)	.18	173 (152)	<.001	.04
*HF, ms^2	219 (301)	136 (161)	.40	82 (93)	<.001	.06
*LF: HF	2.7 (2.3)	2.0 (2.7)	.49	2.3 (2.1)	.21	.60
Time domain						
HR, bpm	128 (17)	128 (20)	.92	132 (14)	.30	.37
Mean NN, ms	477 (60)	488 (78)	.55	460 (48)	.22	.13
SDNN, ms	44 (13)	36 (12)	.02	33 (10)	<.001	.27
*SDΔNN, ms	19 (19)	15 (13)	.40	14 (9)	.002	.07
*RMSSD, ms	19 (19)	15 (10)	.31	14 (9)	.01	.09

Data presented as mean (SD) or *median (IQR) for log transformed data using Independent student t-test between groups. LBF: low body fat; HBF: high body fat; aortic IMT, aortic intima-media thickness; LF, low frequency; HF, high frequency; LF: HF, low frequency/high frequency ratio; HR; heart rate, mean NN; mean of N wave to N wave variation normal; SDNN, the mean of the standard deviation of all normal RR intervals; SDΔNN, SD change in NN; RMSSD, square root of the mean squared differences of successive NN intervals.

Table S4. Cardiac structure and function across body fat percentiles.

		Average body fatness (>25th to ≤75th BF%)		LBF (≤10th BF%)	*P* value (LBF vs average body fatness)		HBF (>90th BF%)	*P* value (HBF vs average body fatness)	*P* value *(LBF vs HBF)*
**Indices of cardiac structure**									
	**n**		**n**			**n**			
PDA present n, %	48	0 (0)	23	2 (8.7)	.14[a]	21	1 (4.8)	.30[a]	.54[a]
VSD present n, %	48	1 (2.1)	23	1 (4.3)	.80[a]	21	1 (4.8)	.52[a]	.73[a]
Left ventricle									
Base to apex length, mm	36	27.1 (2.2)	20	25.5 (3.7)	.04	19	29.0 (3.7)	.02	.005
Diameter, mm	36	16.5 (1.4)	20	15.7 (2.7)	.26	19	16.8 (2.2)	.56	.20
Sphericity index	35	1.6 (0.2)	19	1.7 (0.3)	.28	18	1.9 (0.7)	.18	.93
Septal wall thickness/BSA, mm	45	18.4 (2.7)	21	21.1 (3.8)	.007	23	18.6 (4.4)	.88	.04
Posterior wall thickness/BSA, mm	45	15.5 (2.8)	22	18.5 (2.9)	.000	22	14.4 (2.6)	.13	<.001
End-diastolic dimension/BSA, mm	46	80.3 (8.3)	22	86.9 (8.8)	.005	23	77.7 (7.2)	.18	<.001
Relative wall thickness	45	0.42 (.07)	21	0.46 (.07)	.09	22	0.42 (.07)	.63	.06
Right ventricle									
Base to apex length, mm	33	23.4 (2.4)	14	22.4 (2.9)	.25	17	25.3 (3.4)	.02	.02
Diameter, mm	33	13.8 (1.8)	14	13.0 (1.4)	.18	17	14.2 (1.6)	.40	.03
Sphericity index	33	1.7 (0.2)	14	1.7 (0.2)	.48	16	1.8 (0.2)	.11	.20
**Indices of cardiac function**									
Systolic function									
Heart rate	46	135 (22)	20	136 (18)	.87	19	142 (23)	.23	.34
Fractional shortening, %	46	34.6 (7.2)	22	34.3 (5.8)	.83	23	35.6 (6.2)	.60	.47
Left ventricular output, ml/kg/min	46	230 (69)	19	214 (55)	.39	18	203 (61)	.15	.55
Stroke volume, ml/kg	45	1.8 (0.52)	19	1.6 (0.44)	.27	18	1.4 (0.43)	.03	.27
MAPSE, mm	35	7.0 (1.0)	15	6.6 (0.8)	.20	14	7.1 (1.1)	.62	.12
TAPSE, mm	39	8.7 (1.3)	16	8.4 (1.2)	.52	15	9.4 (1.8)	.11	.09
Mitral lateral S′, cm/s	30	5.4 (0.7)	15	5.5 (0.9)	.68	10	5.9 (0.8)	.07	.26
Mitral septal S′, cm/s	36	4.9 (0.6)	15	4.9 (0.6)	.89	9	4.8 (0.6)	.81	.91
Tricuspid S′, cm/s	24	6.2 (0.6)	15	5.9 (0.9)	.21	9	5.9 (0.8)	.30	.95
Diastolic function									
Mitral E wave, cm/s	36	51 (11)	21	51 (13)	.95	15	55 (11)	.21	.34
Mitral A wave, cm/s	36	51 (10)	21	52 (13)	.71	15	55 (13)	.25	.54
Mitral E/A	37	1.0 (0.1)	21	1.0 (0.1)	.45	15	1.0 (0.1)	.53	.36
Tricuspid E wave, cm/s	40	46 (10)	20	43 (12)	.31	15	52 (13)	.11	.05
Tricuspid A wave, cm/s	40	52 (8)	20	52 (9)	.70	15	57 (11)	.10	.08
Tricuspid E/A	40	0.9 (0.1)	20	0.8 (0.1)	.17	15	0.9 (0.1)	.77	.14
IVRT, ms	32	58 (8)	18	54 (9)	.12	13	55 (5.4)	.27	.68
Mitral lateral E′, cm/s	30	6.8 (1.3)	15	6.1 (1.0)	.08	10	6.8 (1.4)	.98	.15
Mitral septal E′, cm/s	36	5.5 (1.0)	15	5.2 (1.0)	.48	9	6.0 (1.7)	.17	.15
Tricuspid E′, cm/s	24	7.0 (1.6)	15	5.9 (1.1)	.02	9	6.7 (1.6)	.54	.21
E/E′ lateral	28	7.3 (2.5)	15	8.2 (2.5)	.28	10	8.1 (1.8)	.37	.93
E/E′ septal	31	9.1 (2.6)	15	9.5 (2.9)	.64	8	9.3 (2.1)	.88	.84

Data presented as mean (SD). Frequency data No. (%). All cardiac structure and function *P* values adjusted for sex and gestational age from multivariable models. [a] Fisher's Exact test. PDA: patent ductus arteriosus; VSD: ventricular septal defect; BSA: body surface area. MAPSE: Mitral annular plane systolic excursion; TAPSE: Tricuspid annular plane systolic excursion; IVRT: Isovolumetric relaxation time.

Table S5. Indices of cardiac function in infants with low, high and average body fatness.

	Low BF *(relative to Average BF)*		High BF *(Relative to Average BF)*		High BF *(Relative to Low BF)*	
	**β (95% CI)**	***P* value**	**β (95% CI)**	***P* value**	**β (95% CI)**	***P* value**
Systolic function	n=20		n=19			
Heart rate	0.8 (-11.0, 12.0)	.90	7.6 (-4.0, 19.0)	.21	7.8 (-5.8, 21.3)	.25
Fractional shortening, %	-0.2 (-3.8, 3.3)	.70	0.9 (-2.5, 4.4)	.60	1.36 (-2.4, 5.1)	.47
Left ventricular output, ml/kg/min	-11 (-46, 25)	.56	-25 (-60, 11)	.17	-15.0 (-54.7, 24.8)	.45
Stroke volume, ml/kg	-0.1 (-0.4, 0.1)	.33	-0.3 (-0.6, -0.0)	.03	-0.2 (-0.5, 0.1)	.19
MAPSE, mm	-0.4 (-1.0, 0.3)	.29	0.0 (-0.1, 0.1)	.56	0.6 (-0.2, 1.4)	.14
TAPSE, mm	-0.1 (-0.1, 0.1)	.77	0.1 (-0.0, 0.2)	.07	0.7 (-0.2, 2.0)	.12
Mitral lateral S′, cm/s	0.0 (-0.5, 0.5)	.94	0.4 (-0.1, 1.0)	.12	0.4 (-0.3, 1.0)	.27
Mitral septal S′, cm/s	0.0 (-0.4, 0.4)	.98	-0.7 (-0.5, 0.4)	.78	-0.02 (-0.6, 0.5)	.94
Tricuspid S′, cm/s	-0.2 (-0.7, 0.2)	.34	-0.3 (-0.9, 0.3)	.28	-0.1 (-0.8, 0.6)	.69
Diastolic function	n=21		n=15			
Mitral E wave, cm/s	0.9 (-5.4, 7.2)	.78	4.5 (-2.5, 11.5)	.20	3.7 (-4.9, 12.4)	.38
Mitral A wave, cm/s	1.9 (-4.2, 8.0)	.54	4.2 (-2.6, 11.0)	.22	2.3 (-6.5, 11.1)	.60
Mitral E:A	-8.8 (-27.0, 9.0)	.34	-8.2 (-28.0, 12.0)	.42	0.03 (-0.04, 0.1)	.34
Tricuspid E wave, cm/s	-2.5 (-8.8, 3.8)	.43	6.1 (-0.8, 13.0)	.08	9.1 (0.7, 17.5)	.03
Tricuspid A wave, cm/s	-0.5 (-5.6, 4.6)	.85	5.9 (0.3, 11.5)	.04	7.0 (0.4, 13.6)	.04
Tricuspid E:A	-0.1 (-0.1, 0.2)	.19	0.0 (-0.1, 0.1)	.72	0.06 (-0.02, 0.1)	.16
Left isovolumic relaxation time, ms	-3.6 (-8.7, 1.0)	.12	-2.7 (-8.0, 2.7)	.32	1.1 (-5.0, 7.3)	.71
Mitral lateral E′, cm/s	-0.7 (-1.5, 1.5)	.11	0.0 (-1.0, 1.0)	.99	0.7 (-0.3, 1.9)	.17
Mitral septal E′, cm/s	-0.1 (-1.0, 0.6)	.75	0.6 (-0.2, 1.5)	.14	0.7 (-0.5, 1.9)	.23
Tricuspid E′, cm/s	-1.1 (-2.0, -0.1)	.04	-0.5 (-1.6, 0.7)	.43	0.3 (-0.7, 1.3)	.51
E:E′ lateral	0.8 (-0.7, 2.3)	.29	0.9 (-0.8, 2.7)	.29	-0.0 (-2.0, 2.0)	.97
E:E′ septal	0.3 (-1.4, 2.0)	.73	0.3 (-1.8, 2.4)	.80	0.0 (-2.5, 2.6)	.97

Values are unstandardized β-regression coefficients (95% CI) from multivariable models, adjusted for sex and gestational age. Average body fatness; n=46 (systolic function), n=37 (diastolic function). LBF: low body fat; HBF: high body fat; MAPSE, mitral annular peak systolic excursion: TAPSE, tricuspid peak annular.

Reference

1. Haycock GB, Schwartz GJ and Wisotsky DH. Geometric method for measuring body surface area: A height-weight formula validated in infants, children, and adults. The Journal of Pediatrics. 1978;93:62-66.
